# Effectiveness of Mobile Phone and Web-Based Interventions for Diabetes and Obesity Among African American and Hispanic Adults in the United States: Systematic Review

**DOI:** 10.2196/25890

**Published:** 2022-02-04

**Authors:** Chineme Enyioha, Matthew Hall, Christiane Voisin, Daniel Jonas

**Affiliations:** 1 Department of Family Medicine University of North Carolina at Chapel Hill Chapel Hill, NC United States; 2 Cecil G Sheps Center for Health Services Research University of North Carolina at Chapel Hill Chapel Hill, NC United States; 3 Department of Internal Medicine The Ohio State University Columbus, OH United States

**Keywords:** mHealth, mobile health, technology, diabetes, obesity, African American, Hispanic

## Abstract

**Background:**

Mobile health (mHealth) and web-based technological advances allow for new approaches to deliver behavioral interventions for chronic diseases such as obesity and diabetes. African American and Hispanic adults experience a disproportionate burden of major chronic diseases.

**Objective:**

This paper reviews the evidence for mHealth and web-based interventions for diabetes and obesity in African American and Hispanic adults.

**Methods:**

Literature searches of PubMed/Medline, The Cochrane Library, EMBASE, CINAHL Plus, Global Health, Scopus, and Library & Information Science Source were conducted for relevant English-language articles. Articles identified through searches were reviewed by 2 investigators and, if they met the inclusion criteria, were extracted and assessed for risk of bias. Findings were summarized in tabular and narrative format. The overall strength of the evidence was assessed as high, moderate, low, or insufficient on the basis of risk of bias, consistency of findings, directness, precision, and other limitations.

**Results:**

Searches yielded 2358 electronic publications, 196 reports were found to be eligible for inclusion, and 7 studies met the eligibility criteria. All 7 included studies were randomized control trials. Five studies evaluated the effectiveness of an mHealth intervention for weight loss, including one that evaluated the effectiveness for diabetes and two studies focused on diabetes. Of all the studies that focused on weight loss, 3 reported significant differences in weight loss in participants in the intervention group compared with those in the usual care group. Although all studies on diabetes control showed greater improvement in glycemic control for the intervention group compared to that in the control group, only one study showed a significant difference between the 2 groups.

**Conclusions:**

This analysis indicates that there are few published studies that assessed mHealth interventions among minority populations and focused on weight or diabetes. Although the overall strength of evidence was low for diabetes control, it was moderate for weight loss, and our findings suggest that mHealth and web-based interventions may provide a promising approach for interventions among African American and Hispanic adults who have obesity or diabetes.

## Introduction

Mobile health (mHealth) and web-based technological advances allow for new approaches to delivering behavioral interventions for chronic diseases such as obesity and diabetes [1,2]. These are tailored interventions that supplement or augment in-person contact or office visits through activities such as text messaging, the use of downloadable apps with real time feedback, and other mobile technologies [3,4]. mHealth and web-based interventions are easily accessible because a large proportion of Americans have access to a mobile phone and the internet [5,6].

African American and Hispanic adults experience a disproportionate burden of major chronic diseases, have a high prevalence of diabetes and obesity, and end up with worse health outcomes compared to their White counterparts [7]. For instance, African American adults are at a higher risk of obesity than any other racial or ethnic groups, and they are more likely to have uncontrolled diabetes, develop diabetic retinopathy and end stage renal disease, and have a higher diabetes-related mortality rate than non-Hispanic White adults [8-10]. The management of these health conditions can be challenging for patients since a high level of health literacy and numeracy is required for the self-administration of insulin doses, medications, and the maintenance of a healthy diet. This is compounded with limited access to care or very brief clinic visits with limited time for engagement to fully discuss and understand the disease process or treatment plan [11].

mHealth and web-based technology can mitigate some of these access issues and treatment challenges by providing pertinent information for patients with regard to their condition and offering elements such as monitoring of blood glucose levels and insulin dose adjustment, carbohydrate and calorie counting, and physical activities, which can be incorporated into their routine to improve outcomes [11]. Several studies on mHealth and web-based technology have shown promise in the management of diabetes and obesity [12-14]. A number of systematic reviews have suggested significant benefits of mobile or web-based interventions that target physical activity, diet, and diabetes [15-17]. However, these reviews did not specifically focus on underrepresented racial and ethnic minorities such as African American or Hispanic individuals [18-20]. One systematic review that examined participation of African American adults in mHealth interventions focused on recruitment and retention strategies [21]. Another systematic review that examined the effect of health information technology–based diabetes self-management education interventions on medically underserved patients with diabetes included a broader range of technology including computer software with no internet and telemedicine [22].

Furthermore, minority populations such as African American adults are known to be underrepresented in mHealth research despite existing health disparities [21]. mHealth and web-based technology interventions have the potential to reach patients who may have significant barriers to accessing health care the traditional way. A large proportion of underserved minority populations have ownership of a smartphone and access the internet on their mobile device on a daily basis [23]. African American and Hispanic adults, compared to non-Hispanic White adults, have been shown to use mobile phones and text messages for a wider range of functions such as purchasing products, using social network sites, and web-based banking [24], which suggests that they may be more receptive to mHealth interventions, thus bridging the health care access divide.

While a growing body of literature supports the ease and effectiveness of mHealth and web-based interventions for diabetes and obesity, to our knowledge, no systematic review has evaluated the effectiveness of such interventions for improving health outcomes of African American and Hispanic patients. The objective of this study is to provide a review of the evidence for mHealth and web-based interventions for diabetes and obesity in African American and Hispanic patients.

## Methods

### Search Strategy and Data Sources

Our literature search was conducted in accordance with the PRISMA (Preferred Reporting Items for Systematic Reviews and Meta-Analyses) guidelines [25]. We searched for published literature in electronic literature databases PubMed/Medline, The Cochrane Library, EMBASE, CINAHL Plus, Web of Science, Global Health, Scopus, and Library & Information Science Source for unpublished studies, using ClinicalTrials.gov.

A research librarian performed searches for English-language articles on human adults in scientific journals (aged ≥18 years), which combined terms for (1) minority population terms, (2) mHealth intervention terms, and (3) chronic condition terms for type 2 diabetes mellitus and obesity. The last search was conducted on September 23, 2020. Searches were limited by study design to controlled trials. Multimedia Appendix 1 provides full details regarding the search terms. Studies were included if they met the eligibility criteria (Table 1). Selected studies were either randomized controlled trials or nonrandomized controlled trials, and study participants had to be either African American or Hispanic individuals in the United States. Studies were also included if at least 30% of participants were African American adults, Hispanic adults, or a mixture of both minority groups. The intervention was any mobile or web-based health intervention including cellular phone calls, text messaging (SMS or MMS), and web-based applications or downloadable mobile apps that targeted management for diabetes and obesity.

Studies with any of the aforementioned interventions in combination with other types of activities such as keeping a journal of meals or extra group or one-on-one coaching were also included. Studies that focused on patients with diabetes or overweight with other health conditions as a group such as stroke, pregnancy, or depression were excluded from this study. There was no limit on the publication time, duration of the intervention, or participants’ age. For this review, primary outcomes of interest were objective measures related to obesity and diabetes, including BMI, weight change, waist circumference, or hemoglobin A_1c_, which is a standard measure of glycemic control in patients with diabetes.

**Table 1:** Eligibility criteria.

Category	Include	Exclude
Population	Studies focused on African American or Hispanic adults and studies in which African American or Hispanic adults comprise ≥30% of the study population. All age groups.	Studies in which <30% of the population comprises African American or Hispanic adults and special populations such as pregnant women.
Intervention	Controlled trials with any mobile phone and web-based intervention including text messages (SMS or MMS), downloadable apps, use of other hand-held devices, or the internet. Duration of the intervention, frequency of contact, time of the day, expected response or action from participants with each contact such as note or log recording or reply to text message.	N/A^a^
Comparators	Usual care including face-to-face coaching, handouts, and no intervention	Comparative effectiveness studies or studies in which both the intervention and control groups had any form of mobile phone or web-based intervention. Studies in which treatment and control groups differed by other interventions besides the intervention delivered by mobile phones or the web (for instance, the intervention group also attends a group class and control group receives phone calls)
Outcome(s)	Hemoglobin A_1c_, BMI, weight, and waist circumference.	N/A
Timing	No limit	N/A
Setting	Studies performed in the United States	Studies performed in other countries
Study Designs	Randomized and nonrandomized controlled trials (includes pilot studies)	Cohort studies, case-control studies, case series, and meta-analyses

^a^N/A: not applicable.

### Study Selection and Data Extraction

Two reviewers (CE and MH) extracted the data, and each reviewer independently extracting data from all studies. Extracted elements included the following: publication date, authors, study aims and objectives, study design, number of participants in the intervention and control arms, components and duration of the intervention, and results of the study. Outcome measures were extracted at all timepoints for studies that included multiple assessments. Reviewers checked each other’s extractions for accuracy and completeness. Discrepancies were discussed until arrival at an agreement and when necessary, a third reviewer was involved.

### Risk of Bias Assessment, Data Synthesis, and Analysis

Each included study was assessed for risk of bias, using the Cochrane tool for risk of bias in randomized trials [26]. The following domains were assessed for risk of bias: selection bias (including the method of randomization and allocation concealment), detection bias, attrition bias, and reporting bias. Studies were rated as low risk, high risk, or unclear risk. Unclear risk of bias was assigned to a study if there was uncertainty or lack of information.

We summarized our findings for each question in tabular and narrative format. We did not conduct a meta-analysis owing to the limited number of studies that met the inclusion criteria. The overall strength of the evidence (SOE) was assessed as high, moderate, low, or insufficient on the basis of the overall risk of bias of included studies, the consistency of findings across studies, directness, precision, and other limitations [27]. 

## Results

### Search Strategy and Data Sources

**Figure 1:**
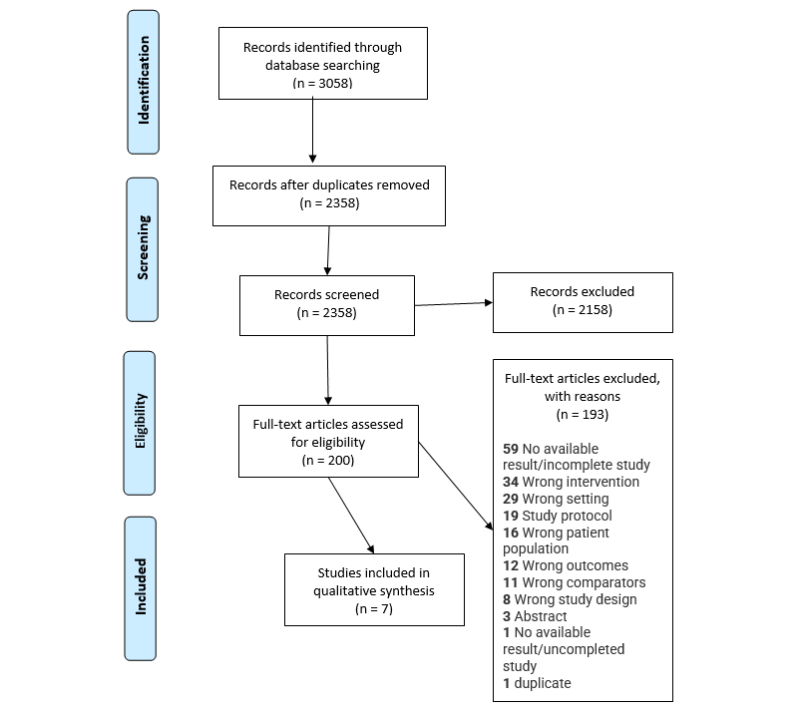
PRISMA (Preferred Reporting Items for Systematic Reviews and Meta-Analyses) flowchart for study selection.

The combined search strategies yielded 2358 electronic citations, which were screened to assess for eligibility (Figure 1). In total, 200 studies were found to be potentially eligible, and their full texts were obtained for further assessment. Of these, 7 studies met the eligibility criteria. The top 4 reasons for rejecting other studies were primarily unavailability of results or incompleteness of studies, incorrect patient population, incorrect intervention, and incorrect setting.

### Study Selection and Data Extraction

The primary aim of 5 studies was to evaluate the effectiveness of an mHealth intervention for weight loss or diabetes, while 2 studies included an evaluation of the feasibility and acceptability of the mHealth intervention as their primary aim. All included studies were randomized control trials [1,2,12,28-31]. Included studies focused on diabetes control (2 trials) [12,28], obesity and weight loss (4 trials) [2,29-31], or both diabetes and obesity (1 trial) [1].

The total number of participants in all studies was 942, with sample sizes ranging from 18 to 371. Three studies had all African American or Hispanic participants, and the other 3 studies had greater than 30% of participants who identified as African American or Hispanic minorities. Study durations ranged from 3 months to 12 months. Mean participant ages across studies ranged from 24 to 53 years. BMI of the participants ranged from 31.5 to 38.0. Mean hemoglobin A_1c_ values for the diabetes studies ranged from 9.02% to 10.2%.

All studies had mobile phones and SMS text messaging as the main mHealth device and medium of communication (Table 2). SMS text messages were sent to participants anywhere from 2-3 times a day to 3-4 times a week. Mobile technology was applied in several ways including medication reminders, prompts for blood glucose check, provision of motivational messages, education on healthy eating patterns, eating cues, and links to other resources and social support.

**Table 2:** Details of intervention and control activities for the included studies.

Study	Study aim	Intervention	Control
Agboola, 2016 [12]	To evaluate the effectiveness on sending daily physical activity–focused text messages in patients with diabetes compared with no text message on physical activity. Furthermore, an evaluation of the effectiveness of the intervention on hemoglobin A_1c_ level, weight change, physical activity, engagement, usability, and satisfaction with the intervention	Participants received a minimum of two automated text messages per day in addition to pedometers—one message in the morning and the other in the evening for 6 months. Messages provided coaching that was dependent on pedometer-captured step counts and physical activity goals target set during the initial visit. Messages received in the morning gave feedback based on activities of the previous day, and messages on other times of the day focused on different coaching themes. Some messages were interactive and focused on elements such as food intake, health status, physical activity, and satisfaction with the program	Pedometers
Arora, 2014 [28]	To evaluate an mHealth intervention for resource-poor emergency department patients with diabetes	Patients received 2 SMS text messages delivered at 9 AM and 5 PM to their mobile phones daily for 6 months. Messages of four categories were sent: one educational/motivational message per day, 3 medication reminders per week, 2 healthy living challenges per week, and 2 trivia messages per week and sent out in question form with the answer sent out an hour later	Usual care (details not provided)
Fortmann, 2017 [1]	To investigate the glycemic effectiveness of a culturally tailored SMS-based diabetes self-management education and support intervention (Dulce Digital)	Participants received information on how to receive and send SMS text messages. Those without a mobile phone were given one. Content of text messages were derived from culturally appropriate Diabetes Self-Management Education and Support curriculum. Participants received 2-3 messages a day initially, which was tapered over 6 months. They received ongoing motivational messages, medication reminders, and prompts for blood glucose measurement	Usual care, which includes visits with a primary care physician, certified diabetes educator, and group diabetes self-management education, dependent on patient or provider initiative
Herring, 2014 [29]	To examine the feasibility, acceptability, and initial efficacy of a technology-based weight loss intervention for urban low-income mothers	Six behavioral health strategies were implemented, one at a time for 2-4 weeks. Participants set realistic goals for each strategy and received 15 minutes biweekly calls from a health counselor. They also receive 3-4 text messages weekly, which probed into their adherence with set goals. Participants also received membership to a Facebook group, which provided access to social support and videos/websites for additional resources.	Regular postpartum care, which is typically one visit with their primary care provider or with a provider through the Special Supplemental Nutrition Program for Women, Infants, and Children (WIC). This visit is usually between 6 and 8 weeks post partum and involves counseling on lactation, birth control, and depression screening. Participants in the study also received counseling on nutrition and vouchers for food and beverages through WIC.
Lin, 2015 [2]	To investigate a behavioral theory-based mobile health intervention to enhance weight loss in patients with obesity	Automated SMS text message program tailored to participants’ selection of 3 relevant goals out of 8 options. Messages were customized to participants’ wake, lunch, and sleep times	Initial assessment including 20-minute individual sessions with a dietician, health status review with a study physician, and receiving educational material on diet and activity. They also received a digital pedometer
Phelan, 2017 [30]	To evaluate the effect of an internet-based weight loss program in addition to the WIC program on weight loss for low-income postpartum women	Internet-based weight loss program with setting of caloric and physical activity goals. Provision of weekly lessons, web diary, weight and physical activity tracker, and instructional and inspirational videos. Participants received 4 SMS text messages per week with notification of new website content and provision of motivation, support, and feedback. This was in addition to all elements of the WIC program	Participants received all aspects of the standard WIC program and a newsletter every 2 months with information on exercise, nutrition, and wellness
Steinberg, 2013 [31]	To evaluate the feasibility of daily text messages for self-monitoring behavioral goals for weight loss among African American women with obesity	Shape plan-tracking of tailored behavior change goals through SMS text messaging. Daily feedback through SMS text messages and weekly feedback by email. At 3 months, participants received skills training information including healthy eating patterns and eating cues to reduce face-to-face contact. At 6 months, a 1-hour face-to-face session that focused on problem-solving, progress assessment, and behavior change	Participants received a health education lesson at the start of the study and at 6 months. They also received a set of videos covering topics on healthy eating and exercise at 3 months, in addition to a pedometer and a prescription to walk 10,000 steps a day

mHealth technology was also used to check on adherence to or progress on set physical activity and dietary goals.

In addition to SMS text messages, 3 studies that focused on obesity had additional activities as part of the intervention. One study included all elements of the Women, Infants, and Children (WIC) program [30], another included 15 minutes biweekly calls from health counselors [29], while the other had a face-to-face 1-hour session at the end of the study, which focused on problem-solving, progress assessment, and behavior change [31].

Obesity trials reported mean weight loss and weight change from baseline, percentage weight loss, and change in BMI. One study reported the proportion of participants who achieved a ≥5% weight loss and a ≥10% weight loss from baseline. The diabetes trial reported hemoglobin A_1c_, weight, and BMI of participants at 3 months and 6 months.

### Risk of Bias Assessment and Data Synthesis and Analysis

With regard to the risk of bias of studies included, a randomization sequence generation process was reported by all studies. Bias for allocation concealment and selective outcome reporting were determined to be low for all studies. Only two studies provided information on blinding of personnel and outcome assessors [2,30]. One trial was determined to have a high risk of bias for incomplete data (up to 63.7% missing at 3 months and 41.1% at 6 months after the start of the intervention) [2,29] and another study was unable to assess differences in provider contact time between intervention and control groups [29].

Overall, 3 studies reported significant differences in weight loss between participants in the intervention group and those in the usual care group [2,29,30] (Table 3). Improvement in weight was noted at 3 months, 14 weeks, 6 months, and 12 months. Two studies found no significant difference in weight loss between groups [1,31].

For the studies that focused on diabetes, all reported an improvement in glycemic control in participants in intervention groups compared to those in control groups [1,12,28], but only one recorded a significant improvement at 6 months [1].

Overall, some evidence supports the efficacy of mHealth and web-based interventions for weight loss among obese African American and Hispanic adults (moderate SOE; Table 4).

**Table 3:** Outcomes of the included studies.

Study participants and sample sizes			Outcome		Baseline data		Result		*P* value
**Agboola, 2016 [12]**									
	126 English- or Spanish-speaking adults with type 2 diabetes and a hemoglobin A_1c_ value of >7.0%	Hemoglobin A_1c_ value at 6 months (SD)		Control: mean hemoglobin A_1c_ 8.38% (SD 1.37%) Intervention: mean hemoglobin A_1c_ 9.02% (SD 1.63%)		Control: mean hemoglobin A_1c_ 8.17% (SD 1.6%) Intervention: mean hemoglobin A_1c_ 8.59% (SD 1.6%)		.14	
**Arora, 2014 [28]**									
	128 adults with poorly controlled diabetes	Median change in hemoglobin A_1c_ at 6 months (95% CI)		Control: mean hemoglobin A_1c_ 10.0% (SD 1.7%) Intervention: mean hemoglobin A_1c_ 10.2% (SD 1.7%)		Control: median change in hemoglobin A_1c_ –0.60 (95% CI –6.8 to 2.11) Intervention: median change in hemoglobin A_1c_ –1.05 (95% CI –5.9 to 2.8)		.23	
**Fortmann, 2017 [1]**									
	126 Hispanic adults with poor glycemic control	Mean hemoglobin A_1c_ at 3 months and 6 months (SD) Mean weight (lbs) and BMI at 3 months and 6 months (SD) Mean BMI (kg/m^2^) at 3 months and 6 months (SD)		Control: mean hemoglobin A_1c_ 9.6% (SD 1.4%) Intervention: mean hemoglobin A_1c_ 9.5% (SD 1.2%) Control: mean weight 176.4 (SD 41.6) lbs Intervention: mean weight 173.1 (SD 34.6) lbs Control: mean BMI 32.2 (SD 6.6) Intervention: mean BMI 31.5 (SD 6.0)		3 months Control: mean hemoglobin A_1c_ 9.3% (SD 1.9%) Intervention: mean hemoglobin A_1c_ 8.5% (SD 1.2%) 6 months Control: mean hemoglobin A_1c_ 9.4% (SD 2.0%) Intervention: mean hemoglobin A_1c_ 8.5% (SD 1.2%) 3 months Control: mean weight 174.2 (SD 39.7) lbs Intervention: mean weight 176.2 (SD 33.0) lbs 6 months Control: mean weight 175.2 (SD 41.6) lbs Intervention: mean weight 174.1 (SD 27.8) lbs 3 months Control: mean BMI 32 (SD 6.1) Intervention: mean BMI 31.7 (SD 5.2) 6 months Control: mean BMI 32.1 (SD 6.6) Intervention: mean BMI 31.9 (SD 5.4)		.03	
**Herring 2014 [29]**									
	18 adult women with singleton infants delivered within the last 2 weeks to 12 months	Mean weight loss (kg) at 14 weeks (SD)		No baseline weight data		Control: mean weight loss 0.5 (SD 2.3) kg Intervention: mean weight loss 2.9 (SD 3.6) kg		.04	
**Lin, 2015 [2]**									
	124 African Americans adults	Mean weight loss (kg) from baseline (95% CI)		Control: mean weight loss 101.2 kg (95% CI 95.7 kg to 106.7 kg) Intervention: mean weight loss 101.8 kg (95% CI 96.4 kg to 107.2 kg)		3 months Control: mean weight loss –0.2 kg (95% CI –1.0 kg to 0.7 kg) Intervention: mean weight loss –2.6 kg (95% CI –3.8 kg to –1.5 kg) 6 months Control: mean weight loss –0.2 kg (95% CI –1.4 kg to 1.0 kg) Intervention: mean weight loss –3.7 kg (95% CI –5.3 kg to –2.1 kg)		<.001 (3 months); .001 (6 months)	
**Phela, 2017 [30]**									
	371 Hispanic adult women monitored 6 weeks to 12 months post partum	Mean weight change (kg) at 6 and 12 months (95% CI)		Control: mean weight change 82.4 kg (95% CI 77.9 kg to 87.1 kg) Intervention: mean weight change 82.5 kg (95% CI 77.5 kg to 87.5 kg)		6 months Control: mean weight change –1.0 kg (95% CI –1.8 kg to –0.2 kg) Intervention: mean weight change –3.1 kg (95% CI –4.0 kg to –2.3 kg) 12 months Control: mean weight change –0.9 kg (95% CI –1.7 kg to –0.1 kg) Intervention: mean weight change –3.2 kg (95% CI –4.1 kg to –2.4 kg)		<.001	
**Steinberg, 2013 [31]**									
	50 African American women with obesity	Mean weight change (kg) at 6 months (SD) Mean change in BMI (kg/m^2^) (SD)		Control: mean weight change 96.0 (SD 23.1) kg Intervention: mean weight change 102.0 (SD 16.6) kg Control: mean BMI change 34.6 (SD 5.8) Intervention: mean BMI change 36.9 (SD 6.2)		Control: mean weight change 1.14 (SD 2.53) kg Intervention: mean weight change –1.27 (SD 6.51) kg Control: mean BMI change 0.42 (SD 0.90) Intervention: mean BMI change –0.47 (SD 2.42)		.09	

**Table 4:** Overall strength of the evidence.

Outcome	Studies (observations), n	Summary of findings	Consistency, directness, and precision	Limitations (including reporting bias)	Overall strength of evidence	Applicability
Weight	5 [1,2,29-31] (689)	Increased weight loss noted in all intervention groups but significant in only 3 studies. One high-quality study [30] (n=371) reported a significant difference in weight change between participants in the intervention and those in the control group	Consistent, indirect, and precise	Two studies had a high risk of bias for nonblinding of study personnel, participants, and outcome assessors. One study had a high risk of bias for incomplete data	Moderate	African American and Hispanic adults with obesity or morbid obesity, who are young and middle-aged adults, and have access to a mobile phone
Hemoglobin A_1c_	3 [1,12,28] (380)	Improvement in hemoglobin A_1c_ in the intervention groups but only significant in one study	Consistent, indirect, and imprecise	Studies had a high risk of bias from the nonblinding of study personnel and participants. One study also had a high risk of bias from the nonblinding of outcome assessors and the other study had an unclear risk of bias for the same	Low	African American or Hispanic adults with poorly controlled diabetes and access to a mobile phone

## Discussion

### Principal Findings

We identified 7 trials that reported the benefit of mHealth interventions directed toward obesity and diabetes among African American and Hispanic adults. We found an association between receiving the mHealth intervention and weight loss compared to those in control groups, which suggests that mHealth and web-based interventions are effective for minority patients who are overweight. Of the three studies that addressed diabetes, only one reported an improvement in hemoglobin A_1c_ among those who received mHealth interventions compared to their control group counterparts, suggesting that the evidence may be unclear for the role of mHealth interventions in diabetes control among patients in ethnic minorities. This systematic review shows that mHealth and web-based interventions for 6 months to a year can be effective for weight loss in African American and Hispanic adults.

Our findings in support of mHealth interventions for weight loss among patients in ethnic minorities are similar to those of other studies [32-34]. A systematic review by Ryan et al [32] reported that mHealth intervention for weight loss is beneficial, while the systematic review and meta-analysis by Sorgente et al [33] was more specific in suggesting that web-based interventions are more effective than usual care or minimal intervention, but the evidence is conflicting with regard to the benefit of web-based interventions compared to non–web-based interventions. In our systematic review, of the 3 studies that reported significant weight loss in the intervention group, one of them had a control group that received usual care, while control group participants in the other 2 studies had comparable non-mHealth interventions. Beleigoli et al [34] suggest that web-based interventions only lead to a short-term weight loss but provide no evidence for weight loss over a long period of time. The longest study duration in our review was 12 months. Studies beyond 12 months, which met our inclusion criteria, were not found, and it is unclear if participants will remain in an intervention of this kind for longer than a period of a year. Changes in diet and activity patterns during holiday periods, the challenge or burden of adherence to self-monitoring over a long period of time, and the need for coaching in addition to self-monitoring have been suggested as reasons for the difficulty in maintaining the long-term effects of mHealth interventions for obesity and weight loss [35,36].

We also observed an improvement in hemoglobin A_1c_ with mHealth interventions compared to those with usual care. Our findings are similar to those of other studies that have shown improved glycemic control with mHealth interventions in the general population [12,14,36], except that only one study in this review showed a significant difference at 6 months. Another systematic review showed an improvement in 42% of studies in the review [37]. One major finding noted in the diabetes studies in this review was that the study with significant results incorporated culturally appropriate messages as part of their intervention [1]. Even with the improvements noted, it is unclear if outcomes would be significantly better if mHealth and web-based interventions have improved usability. African American and Hispanic adults have been found to be less adherent to interventions with technology [38,39]. Better outcomes have been noted in studies where the intervention was developed to be more appealing to the target minority population [40]. The lack of cultural consistency and application has been noted as a major challenge with mHealth and web-based interventions, which can influence results and outcomes [37,41]. Furthermore, some studies have shown that diabetes mHealth programs do not always meet health literacy guidelines, and they are not as user-friendly [42]. The design of some mHealth apps makes it challenging for some groups of people. For instance, Hispanic patients with diabetes who use apps are more likely to use certain functionalities such as medication and blood glucose diaries and less likely to use diaries developed for insulin and hemoglobin A_1c_ [43].

Another important finding from this study is the limited number of studies with African American and Hispanic adults in mHealth interventions for obesity and diabetes. The underrepresentation of participants from minority groups in research studies has been well-documented [44-46] and is reflective in this review. Several reasons have been cited for this, including barriers related to misconceptions or lack of information, mistrust, concerns about outcomes, stigmatization, insurance coverage, and immigration status in the United States [46-48]. More studies focused on mHealth interventions for African American and Hispanic adults with diabetes and obesity will augment the current evidence on the role of mHealth interventions for chronic disease management. This may also contribute to reducing the disparities gap that currently exist in the health care system.

### Limitations

The limited number of studies with primarily African American and Hispanic participants is a limitation of this study, which has also been observed in several other studies [21]. Another limitation is our study inclusion criteria. This review focused on African American and Hispanic participants and only included studies with ≥30% of the participants of interest. The team considered a set of less rigid inclusion criteria but decided against it since greater the proportion of African American or Hispanic participants, greater the ability to make conclusions specifically about the effectiveness of mHealth interventions on these racial groups in these studies. While all included studies involved mobile phones and SMS text messages, they differed in the content of the intervention, sample sizes, and study duration. The absence of studies with data beyond 12 months is also a limitation. Obesity and diabetes are chronic conditions that require long-term management and lifestyle modification. Furthermore, although some studies that met our inclusion criteria showed significant changes, it is unclear whether these translate to clinical significance.

### Conclusions

Although the overall strength of the evidence was moderate for weight loss and low for diabetes, our findings suggest that mHealth and web-based interventions may provide a promising approach. mHealth technology can be used to meet different health needs such as the delivery of motivational messages, health education, prompts or reminders, and personal coaching [49]. Further studies are required to fully understand the role of mHealth interventions in chronic disease management in minority patients. These findings are significant because minority patients bear a heavy burden of diabetes and obesity in the United States. mHealth and web-based interventions have the potential to assist medical providers as they provide care to patients with chronic disease and improve health disparities. Further research to better understand how mHealth and web-based interventions can help overcome challenges or barriers to chronic disease management and will be very useful.

## Appendix

Multimedia Appendix 1 Search terms.

## Abbreviations

mHealth: mobile health
SOE: strength of the evidence
WIC: Women, Infants, and Children
