# Supplementary material for: Effectiveness of Mobile Phone and Web-Based Interventions for Diabetes and Obesity Among African American and Hispanic Adults in the United States: Systematic Review
Source: JMIR Public Health Surveill. 2022 Feb 4;8(2):e25890. doi: 10.2196/25890 (PMC8857702; doi:10.2196/25890)
Supplement: Multimedia Appendix 1 [file publichealth_v8i2e25890_app1.pdf]

Search	**PUBMED search**
#1	Search mhealth[tiab] OR "mobile health"[tiab] OR "Telemedicine"[Mesh] OR telemedicine[tiab] OR telehealth[tiab] OR "Text Messaging"[Mesh] OR "text message"[All Fields] OR "text messages"[All Fields] OR "text messaging"[All Fields] OR "electronic health"[tiab]
#2	Search (“Cell Phone"[Mesh] OR "Social Media"[Mesh] OR “mobile phone"[All Fields] OR "mobile phones"[All Fields] OR "cell phone"[All Fields] OR "cellular phone"[All Fields] OR "cell phones"[All Fields] OR "cellular phones"[All Fields])
#3	Search (SMS[tiab] OR “Short messaging system”[All Fields] OR “mobile-based interventions”[All Fields] OR Computers OR “Hand-held devices”[All Fields] OR PDA[tiab] OR “Personal Digital Assistant”[All Fields] OR Tablets OR Apps OR "Mobile Applications"[Mesh] OR "Computers, Handheld"[Mesh] OR “Pocket computer”[All Fields] OR “Handheld computer”[All Fields] OR “Pocket PC”[All Fields] OR "Smartphone"[Mesh] OR Smartphone OR “Smart phone”[All Fields] OR “Smart book”[All Fields] OR Iphone OR I-Phone OR Blackberry OR MMS[tiab] OR “Multimedia messages”[All Fields] OR "Electronic Mail"[Mesh] OR Email OR E-Mail OR “Electronic mail”[All Fields] OR Web-based[tiab] OR “Interactive software”[All Fields] OR “Hand held computer”[All Fields] OR “Ultra mobile”[All Fields] OR "MP3-Player"[Mesh] OR “MP3 player”[All Fields] OR “MP4 player”[All Fields] OR Ipod[tiab])
#4	Search (#1 and #2)
#5	Search (#1 and #3)
#6	Search (#4 or #5)
#7	Search ("Diabetes Mellitus, Type 2"[Mesh] OR “diabetes mellitus type 2”[All Fields] OR “type 2 diabetes mellitus”[All Fields] OR (“diabetes” AND “mellitus” AND “type 2”))
#8	Search (#6 and #7)
#9	Search ("Diet"[Mesh] OR "Diet, Protein-Restricted"[Mesh] OR "Diet, Fat-Restricted"[Mesh] OR "Diet, Reducing"[Mesh] OR "Diet Therapy"[Mesh] OR "Diet, Carbohydrate-Restricted"[Mesh] OR Diet*[tiab] OR "Exercise"[Mesh] OR Exercise*[tiab] OR "Physical Activity"[All Fields] OR "physically active"[All Fields] OR "Food"[Mesh] AND "Functional Food"[Mesh] AND "Food and

	Beverages"[Mesh] AND "Food Industry"[Mesh] AND "Food Quality"[Mesh] OR Food*[tiab] OR "Food Preferences"[Mesh] OR “food preference”[All Fields] OR "Obesity"[Mesh] OR Overweight[Mesh] OR overweight OR obes*[tiab])
#10	Search (#6 and #9)
#11	Search (#8 or #10)
#12	Search (#8 or #10) Filters: Humans
#13	Search (#8 or #10) Filters: Humans; English
#14	Search ((randomized[title/abstract] OR randomised[title/abstract]) AND controlled[title/abstract] AND trial[title/abstract]) OR (controlled[title/abstract] AND trial[title/abstract]) OR "controlled clinical trial"[publication type] OR "Randomized Controlled Trial"[Publication Type] OR "Single-Blind Method"[MeSH] OR "Double-Blind Method"[MeSH] OR "Random Allocation"[MeSH])
#15	Search (#13 and #14)
#16	Search "African Continental Ancestry Group"[Mesh] OR “African Americans”[Mesh] OR Blacks OR “Hispanic Americans”[Mesh] OR “Mexican Americans”[Mesh] OR Latino OR Latina OR “Racial Stocks”[Mesh] OR “Ethnic Groups”[Mesh] OR “Minority Groups”[Mesh] OR “Population Groups”[Mesh] OR “Indians, North American”[Mesh] OR “Native American”[All Fields] OR Tribal* OR “Asian Americans”[Mesh] OR “Asian American”[All Fields] OR divers* OR "Medically Underserved Area"[Mesh] OR underserved OR "Vulnerable Populations"[Mesh] OR disadvantaged OR "Social Class"[Mesh] OR “low socioeconomic status”[All Fields]
#17	Search (#15 and #16)

ID	**Cochrane Library Search**

#1	Mhealth:ti or MHealth:ab or "mobile health":ti or "mobile health":ab or [mh Telemedicine] or telemedicine:ti or telemedicine:ab or telehealth:ti or telehealth:ab or [mh "Text Messaging"] or "text message":ti or "text message":ab or "text messages":ti or "text messages":ab or "text messaging" or "electronic health":ti or "electronic health":ab
#2	[mh "Cell Phone"] or [mh "Social Media"] or "mobile phone" or "mobile phones" or "cell phone" or "cellular phone" or "cell phones" or "cellular phones"
#3	SMS:ti or SMS:ab or "Short messaging system" or "mobile-based interventions" or Computers or "Hand-held devices" or PDA:ti or PDA:ab or "Personal Digital Assistant" or Tablets or Apps or [mh "Mobile Applications"] or [mh "Computers, Handheld"] or "Pocket computer" or "Handheld computer" or "Pocket PC" or [mh Smartphone] or Smartphone or "Smart phone" or "Smart book" or Iphone or I-Phone or Blackberry or MMS:ti or MMS:ab or "Multimedia messages" or [mh "Electronic Mail"] or Email or E-Mail or "Electronic mail" or Web-based:ti or Web-based:ab or "Interactive software" or "Hand held computer" or "Ultra mobile" or [mh MP3-Player] or "MP3 player" or "MP4 player" or Ipod:ti or Ipod:ab
#4	#1 and #2
#5	#1 and #3
#6	#4 or #5
#7	[mh "Diabetes Mellitus, Type 2"] or "diabetes mellitus type 2" or "type 2 diabetes mellitus" or ("diabetes" and "mellitus" and "type 2")
#8	#6 and #7
#9	[mh Diet] or [mh "Diet, Protein-Restricted"] or [mh "Diet, Fat-Restricted"] or [mh "Diet, Reducing"] or [mh "Diet Therapy"] or [mh "Diet, Carbohydrate-Restricted"] or Diet*:ti or Diet*:ab or [mh Exercise] or Exercise*:ti or Exercise*:ab or [mh "Physical Activity"] or "physically active" or [mh Food] and [mh "Functional Food"] and [mh "Food and Beverages"] and [mh "Food Industry"] and [mh "Food Quality"] or Food*:ti or Food*:ab or [mh "Food Preferences"] or "food preference" or [mh Obesity] or [mh Overweight] or overweight or obes*:ti or obes*:ab
#10	#6 and #9

#11	#8 or #10
#12	[mh "African Continental Ancestry Group"] or [mh "African Americans"] or Blacks or [mh "Hispanic Americans"] or [mh "Mexican Americans"] or Latino or Latina or [mh "Racial Stocks"] or [mh "Ethnic Groups"] or [mh "Minority Groups"] or [mh "Population Groups"] or [mh "Indians, North American"] or "Native American" or Tribal* or [mh "Asian Americans"] or "Asian American" or divers* or [mh "Medically Underserved Area"] or underserved or [mh "Vulnerable Populations"] or disadvantaged or [mh "Social Class"] or "low socioeconomic status"
#13	#11 and #12
#14	#13 in Cochrane Reviews (Reviews and Protocols), Other Reviews and Trials

#	**EMBASE search**
#1	'telemedicine'/exp OR 'cell phone'/exp OR 'text messaging'/exp OR 'social media'/exp OR mhealth:ab,ti OR 'mobile health':ab,ti OR 'electronic health':ab,ti OR telemedicine:ab,ti OR telehealth:ab,ti OR 'text message' OR 'text messages' OR 'text messaging' OR 'mobile phone' OR 'mobile phones' OR 'cell phone' OR 'cellular phone' OR 'cell phones' OR 'cellular phones'
#2	'electronic mail'/exp OR 'mp3-player'/exp OR 'mobile applications'/exp OR 'computers, handheld'/exp OR 'smartphone'/exp OR sms:ab,ti OR pda:ab,ti OR mms:ab,ti OR ipod:ab,ti OR 'web based':ab,ti OR 'short messaging system' OR 'mobile-based interventions'OR computers OR 'hand-held devices' OR 'personal digital assistant' OR tablets OR apps OR 'pocket computer' OR 'handheld computer' OR 'pocket pc' OR smartphone OR 'smart phone' OR 'smart book' OR iphone OR 'i phone' OR blackberry OR 'multimedia messages' OR email OR 'e mail' OR 'electronic mail' OR 'interactive software' OR 'hand held computer' OR 'ultra mobile' OR 'mp3 player' OR 'mp4 player'
#3	#1 AND #2
#4	'diabetes mellitus, type 2'/exp OR 'diabetes mellitus type 2' OR 'type 2 diabetes mellitus' OR ('diabetes' AND 'mellitus' AND 'type 2')
#5	#3 AND #4
#6	'diet'/exp OR 'diet, protein-restricted'/exp OR 'diet, fat-restricted'/exp OR 'diet, reducing'/exp OR 'diet therapy'/exp OR 'diet, carbohydrate-restricted'/exp OR diet*:ab,ti OR 'exercise'/exp OR exercise*:ab,ti OR 'physical activity' OR 'physically active' OR 'food'/exp OR 'functional food'/exp OR 'food and beverages'/exp OR 'food industry'/exp OR 'food quality'/exp OR food*:ab,ti OR 'food preferences'/exp OR 'food preference' OR 'obesity'/exp OR 'overweight'/exp OR overweight OR obes*:ab,ti
#7	#3 AND #6

#8	#5 OR #7
#9	'randomized controlled trial'/exp OR 'single blind procedure'/exp OR 'double blind procedure'/exp OR 'random allocation'/exp
#10	#8 AND #9
#11	'african continental ancestry group'/exp OR 'african americans'/exp OR blacks OR 'hispanic americans'/exp OR 'mexican americans'/exp OR latino OR latina OR 'racial stocks'/exp OR 'ethnic groups'/exp OR 'minority groups'/exp OR 'population groups'/exp OR 'indians, north american'/exp OR 'native american' OR tribal* OR 'asian americans'/exp OR 'asian american' OR divers* OR 'medically underserved area'/exp OR 'vulnerable populations'/exp OR underserved OR disadvantaged OR 'social class'/exp OR 'low socioeconomic status'
#12	#10 AND #11

CINAHL Plus with Full Text (EBSCOhost)

#	Query	Limiters/Expanders	Results
S1	TI mhealth OR AB mhealth OR TI "mobile health" OR AB "mobile health" OR MH "Telemedicine+" OR TI telemedicine OR AB telemedicine OR TI telehealth OR AB telehealth OR MH "Text Messaging+" OR TX "text message" OR TX "text messages" OR TX "text messaging" OR TI "electronic health" OR AB "electronic health"	Expanders - Apply equivalent subjects<br>Search modes - Boolean/Phrase	39,733
S2	MH "Cellular Phone+" OR MH "Social Media" OR TX "mobile phone" OR TX "mobile phones" OR TX "cell phone" OR TX "cellular phone" OR TX "cell phones" OR TX "cellular phones"	Expanders - Apply equivalent subjects<br>Search modes - Boolean/Phrase	36,315
S3	TI SMS OR AB SMS OR TX "Short messaging system" OR TX "mobile-based interventions" OR Computers OR TX "Hand-held devices" OR TI PDA OR AB PDA OR TX "Personal Digital Assistant" OR Tablets OR Apps OR MH "Mobile Applications" OR MH "Computers, Hand-Held+" OR TX "Pocket computer" OR TX "Handheld computer" OR TX "Pocket PC" OR Smartphone OR TX "Smart phone" OR TX "Smart book" OR Iphone OR I-Phone OR Blackberry OR TI MMS AB MMS OR	Expanders - Apply equivalent subjects<br>Search modes - Boolean/Phrase	203,270

	TX "Multimedia messages" OR MH "Email" OR Email OR E-Mail OR TX "Electronic mail" OR TI "Web-based" OR AB "web-based" OR TX "Interactive software" OR TX "Hand held computer" OR TX "Ultra mobile" OR TX "MP3 player" OR TX "MP4 player" OR TI Ipod OR AB Ipod		
S4	S1 AND S2	Expanders - Apply equivalent subjects<br>Search modes - Boolean/Phrase	6,058
S5	S1 AND S3	Expanders - Apply equivalent subjects<br>Search modes - Boolean/Phrase	8,412
S6	S4 OR S5	Expanders - Apply equivalent subjects<br>Search modes - Boolean/Phrase	12,008
S7	MH "Diabetes Mellitus, Type 2" OR TX "diabetes mellitus type 2" OR TX "type 2 diabetes mellitus" OR ("diabetes" AND "mellitus" AND "type 2")	Expanders - Apply equivalent subjects<br>Search modes - Boolean/Phrase	73,031
S8	S6 AND S7	Expanders - Apply equivalent subjects<br>Search modes - Boolean/Phrase	236
S9	MH "Diet+" OR MH "Diet, Fat-Restricted" OR MH "Restricted Diet+" OR MH "Diet Therapy+" OR MH "Diet, Low Carbohydrate" OR TI Diet* OR AB Diet* OR MH "Exercise+" OR TI Exercise* OR AB Exercise* OR TX "Physical Activity" OR TX "physically active" OR MH "Food+" OR MH "Functional Food" OR MH "Food and Beverages+" OR MH "Food Industry+" OR MH "Food Quality+" OR TI Food* OR AB Food* OR MH "Food Preferences" OR TX "food preference" OR MH "Obesity+" OR overweight OR TI obes* OR AB obes*	Expanders - Apply equivalent subjects<br>Search modes - Boolean/Phrase	693,111

S10	S6 AND S9	Expanders - Apply equivalent subjects<br>Search modes - Boolean/Phrase	1,481
S11	S8 OR S10	Expanders - Apply equivalent subjects<br>Search modes - Boolean/Phrase	1,623
S12	S11	Limiters - English Language; Human<br>Expanders - Apply equivalent subjects<br>Search modes - Boolean/Phrase	1,032
S13	S12	Limiters - Publication Type: Randomized Controlled Trial<br>Expanders - Apply equivalent subjects<br>Search modes - Boolean/Phrase	285
S14	MH "Blacks" OR "African Americans" OR Blacks OR MH "Hispanics" OR TX "Hispanic Americans" OR TX "Mexican Americans" OR Latino OR Latina OR Latinx OR MH "Race Factors" OR MH "Ethnic Groups+" OR MH "Minority Groups" OR TX "Population Groups" OR MH "Native Americans" OR "Native American"[All Fields] OR Tribal* OR MH "Asians+" OR TX "Asian Americans" OR TX "Asian American" OR divers* OR MH "Medically Underserved Area" OR underserved OR MH "Special Populations" OR disadvantaged OR MH "Social Class+" OR MH "Socioeconomic Factors+" OR TX "low socioeconomic status"	Limiters - Publication Type: Randomized Controlled Trial<br>Expanders - Apply equivalent subjects<br>Search modes - Boolean/Phrase	7,331
S15	S13 AND S14	Expanders - Apply equivalent subjects<br>Search modes - Boolean/Phrase	52

Web Of Science

Included databases in search:

Science Citation Index Expanded (SCI-EXPANDED) --1900-present; Social Sciences Citation Index (SSCI) --1900-present; Book Citation Index– Science (BKCI-S) --2005-present; Book Citation Index– Social Sciences & Humanities (BKCI-SSH) --2005-present; and Emerging Sources Citation Index (ESCI) --2015-present

Combined search terms not allowed to exceed 100 terms. Therefore, no racial terms were used to limit, and the diabetes and the diet/exercise/obesity searches limited to reviews and saved separately:

a) Diabetes search: 28 results; 28 imported to EN; then combined with search b below to import to Covidence
b) Diet/exercise/obesity search: 318 results (309 after import to EN); 337 when combined in EN with search a.

335 total new imported to Covidence – most do not look relevant (no minorities/underserved terms in titles)

#1	53,974	ALL=(mhealth OR "mobile health" OR "Telemedicine" OR telehealth OR "Text Messaging" OR "text message" OR "text mess ages" OR "electronic health")<br>*Indexes=SCI-EXPANDED, SSCI, BKCI-S, BKCI-SSH, ESCI Timespan=All years*
#2	73,623	**ALL FIELDS:** ("Cell Phone" OR "Social Media" OR "mobile phone" OR "mobile phones" OR "cellular phone" OR "cell phones" OR "cellular phones")<br>*Indexes=SCI-EXPANDED, SSCI, BKCI-S, BKCI-SSH, ESCI Timespan=All years*
#3	1,421,381	ALL=(SMS OR “Short messaging system” OR “mobile-based interventions” OR Computers OR “Hand-held devices” OR PDA OR “Personal Digital Assistant” OR Tablets OR Apps OR "Mobile Applications" OR Computer* OR “ Pocket PC” OR "Smartphone" OR “Smart phone” OR “Smart book” OR Iphone OR I-Phone OR Blackberry OR MMS OR “Multimedia messages” OR Email OR E-Mail OR “Electronic mail” OR Web-based OR “Interactive software” OR “Ultra mobile” OR “MP3 player” OR “MP4 player” OR Ipod)<br>*Indexes=SCI-EXPANDED, SSCI, BKCI-S, BKCI-SSH, ESCI Timespan=All years*
#4	4,271	#2 AND #1<br>*Indexes=SCI-EXPANDED, SSCI, BKCI-S, BKCI-SSH, ESCI Timespan=All years*
#5	12,420	#3 AND #1<br>*Indexes=SCI-EXPANDED, SSCI, BKCI-S, BKCI-SSH, ESCI Timespan=All years*
#6	14,382	#5 OR #4

		Indexes=SCI-EXPANDED, SSCI, BKCI-S, BKCI-SSH, ESCI Timespan=All years
#7	90,313	**ALL FIELDS:** (("Diabetes Mellitus, Type 2" OR “diabetes mellitus type 2” OR “type 2 diabetes mellitus” OR (“diabetes” AND “mellitus” AND “type 2”)))<br>*Indexes=SCI-EXPANDED, SSCI, BKCI-S, BKCI-SSH, ESCI Timespan=All years*
#8	28	(#7 AND #6) *AND* **DOCUMENT TYPES:** (Review)<br>*Indexes=SCI-EXPANDED, SSCI, BKCI-S, BKCI-SSH, ESCI Timespan=All years*
#9	2,831,576	ALL=("Diet" OR Exercise* OR "Physical Activity" OR "physically active" OR "Food" OR "Obesity" OR Overweight OR obes *)<br>*Indexes=SCI-EXPANDED, SSCI, BKCI-S, BKCI-SSH, ESCI Timespan=All years*
#10	318	(#9 AND #6) *AND* **DOCUMENT TYPES:** (Review)<br>*Indexes=SCI-EXPANDED, SSCI, BKCI-S, BKCI-SSH, ESCI Timespan=All years*

Global Health (EBSCOhost)

#	Query	Limiters/Expanders	Results
S1	TX (mhealth OR "mobile health" OR "Telemedicine" OR telehealth OR "Text Messaging" OR "text message" OR "text messages" OR "electronic health")	Expanders - Apply equivalent subjects Search modes - Boolean/Phrase	5,766
S2	TX ("Cell Phone" OR "Social Media" OR "mobile phone" OR "mobile phones" OR "cellular phone" OR "cell phones" OR "cellular phones")	Expanders - Apply equivalent subjects Search modes - Boolean/Phrase	5,444
S3	TX (SMS OR “Short messaging system” OR “mobile-based interventions” OR Computers OR “Hand-held devices” OR	Expanders - Apply equivalent subjects	52,319

	PDA OR “Personal Digital Assistant” OR Tablets OR Apps OR "Mobile Applications" OR Computer* OR “Pocket PC” OR "Smartphone" OR “Smart phone” OR “Smart book” OR Iphone OR I-Phone OR Blackberry OR MMS OR “Multimedia messages” OR Email OR E-Mail OR “Electronic mail” OR Web-based OR “Interactive software” OR “Ultra mobile” OR “MP3 player” OR “MP4 player” OR Ipod)	Search modes - Boolean/Phrase	
S4	S1 AND S2	Expanders - Apply equivalent subjects Search modes - Boolean/Phrase	793
S5	S1 AND S3	Expanders - Apply equivalent subjects Search modes - Boolean/Phrase	1,360
S6	S4 OR S5	Expanders - Apply equivalent subjects Search modes - Boolean/Phrase	1,793
S7	(("Diabetes Mellitus, Type 2" OR “diabetes mellitus type 2” OR “type 2 diabetes mellitus” OR (“diabetes” AND “mellitus” AND “type 2”)))	Expanders - Apply equivalent subjects	20,470

		Search modes - Boolean/Phrase	
S8	S6 AND S7	Expanders - Apply equivalent subjects Search modes - Boolean/Phrase	17
S9	("Diet" OR Exercise* OR "Physical Activity" OR "physically active" OR "Food" OR "Obesity" OR Overweight OR obes*)	Expanders - Apply equivalent subjects Search modes - Boolean/Phrase	982,364
S10	S6 AND S9	Expanders - Apply equivalent subjects Search modes - Boolean/Phrase	331
S11	S8 OR S10	Expanders - Apply equivalent subjects Search modes - Boolean/Phrase	341
S12	"African Continental Ancestry Group" OR “African Americans” OR Blacks OR “Hispanic Americans” OR “Mexican Americans” OR Latino OR Latina OR Latinx OR “Racial Stocks” OR “Ethnic Groups” OR “Minority Groups” OR “Population Groups” OR “Indians, North American” OR	Expanders - Apply equivalent subjects Search modes - Boolean/Phrase	202,623

	“Native American” OR Tribal* OR “Asian Americans” OR “Asian American” OR divers* OR "Medically Underserved Area" OR underserved OR "Vulnerable Populations" OR disadvantaged OR "Social Class" OR “low socioeconomic status”		
S13	S11 AND S12	Expanders - Apply equivalent subjects Search modes - Boolean/Phrase	47
S14	((randomized OR randomised) AND controlled AND trial) OR (controlled AND trial) OR "controlled clinical trial" OR "Randomized Controlled Trial" OR "Single-Blind Method" OR "Double-Blind Method" OR "Random Allocation"	Expanders - Apply equivalent subjects Search modes - Boolean/Phrase	65,600
S15	S13 AND S14	Expanders - Apply equivalent subjects Search modes - Boolean/Phrase	**16**

Scopus (Elsevier)

History Count	Search Terms	Results
1	TITLE-ABS-KEY ((mhealth OR "mobile health" OR "Telemedicine" OR telehealth OR "Text Messaging" OR "text message" OR "text messages" OR "electronic health"))	106,640 document results
2	TITLE-ABS-KEY (("Cell Phone" OR "Social Media" OR "mobile phone" OR "mobile phones" OR "cellular phone" OR "cell phones" OR "cellular phones"))	159,175 document results

History Count	Search Terms	Results
3	(TITLE-ABS-KEY ((sms OR "Short messaging system" OR "mobile-based interventions" OR computers OR "Hand-held devices" OR pda OR "Personal Digital Assistant" OR tablets OR apps OR "Mobile Applications" OR computer* OR "Pocket PC" OR "Smartphone" OR "Smart phone")) OR TITLE-ABS-KEY (("Smart book" OR iphone OR i-phone OR blackberry OR mms OR "Multimedia messages" OR email OR e-mail OR "Electronic mail" OR web-based OR "Interactive software" OR "Ultra mobile" OR "MP3 player" OR "MP4 player" OR ipod)))	6,466,910 document results
6	(TITLE-ABS-KEY ((mhealth OR "mobile health" OR "Telemedicine" OR telehealth OR "Text Messaging" OR "text message" OR "text messages" OR "electronic health"))) AND ((TITLE-ABS-KEY (("Cell Phone" OR "Social Media" OR "mobile phone" OR "mobile phones" OR "cellular phone" OR "cell phones" OR "cellular phones"))) OR ((TITLE-ABS-KEY ((sms OR "Short messaging system" OR "mobile-based interventions" OR computers OR "Hand-held devices" OR pda OR "Personal Digital Assistant" OR tablets OR apps OR "Mobile Applications" OR computer* OR "Pocket PC" OR "Smartphone" OR "Smart phone")) OR TITLE-ABS-KEY (("Smart book" OR iphone OR i-	41,097 document results

History Count	Search Terms	Results
	phone OR blackberry OR mms OR "Multimedia messages" OR email OR e-mail OR "Electronic mail" OR web-based OR "Interactive software" OR "Ultra mobile" OR "MP3 player" OR "MP4 player" OR ipod)))))	
7	TITLE-ABS-KEY ((("Diabetes Mellitus, Type 2" OR "diabetes mellitus type 2" OR "type 2 diabetes mellitus" OR ("diabetes" AND "mellitus" AND "type 2"))))	180,022 document results
8	((TITLE-ABS-KEY ((mhealth OR "mobile health" OR "Telemedicine" OR telehealth OR "Text Messaging" OR "text message" OR "text messages" OR "electronic health"))) AND ((TITLE-ABS-KEY (("Cell Phone" OR "Social Media" OR "mobile phone" OR "mobile phones" OR "cellular phone" OR "cell phones" OR "cellular phones"))) OR ((TITLE-ABS-KEY ((sms OR "Short messaging system" OR "mobile-	585 document results

History Count	Search Terms	Results
	based interventions" OR computers OR "Hand-held devices" OR pda OR "Personal Digital Assistant" OR tablets OR apps OR "Mobile Applications" OR computer* OR "Pocket PC" OR "Smartphone" OR "Smart phone")) OR TITLE-ABS-KEY (("Smart book" OR iphone OR i-phone OR blackberry OR mms OR "Multimedia messages" OR email OR e-mail OR "Electronic mail" OR web-based OR "Interactive software" OR "Ultra mobile" OR "MP3 player" OR "MP4 player" OR ipod)))))) AND (TITLE-ABS-KEY ((("Diabetes Mellitus, Type 2" OR "diabetes mellitus type 2" OR "type 2 diabetes mellitus" OR ("diabetes" AND "mellitus" AND "type 2")))))	
9	TITLE-ABS-KEY (("Diet" OR exercise* OR "Physical Activity" OR "physically active" OR "Food" OR "Obesity" OR overweight OR obes*))	3,026,820 document results

History Count	Search Terms	Results
12	((TITLE-ABS-KEY ((mhealth OR "mobile health" OR "Telemedicine" OR telehealth OR "Text Messaging" OR "text message" OR "text messages" OR "electronic health"))) AND ((TITLE-ABS-KEY (("Cell Phone" OR "Social Media" OR "mobile phone" OR "mobile phones" OR "cellular phone" OR "cell phones" OR "cellular phones"))) OR ((TITLE-ABS-KEY ((sms OR "Short messaging system" OR "mobile-based interventions" OR computers OR "Hand-held devices" OR pda OR "Personal Digital Assistant" OR tablets OR apps OR "Mobile Applications" OR computer* OR "Pocket PC" OR "Smartphone" OR "Smart phone")) OR TITLE-ABS-KEY (("Smart book" OR iphone OR i-phone OR blackberry OR mms OR "Multimedia messages" OR email OR e-mail OR "Electronic mail" OR web-based OR "Interactive software" OR "Ultra mobile" OR "MP3 player" OR "MP4 player" OR ipod)))))) AND (TITLE-ABS-KEY (("Diet" OR exercise* OR "Physical Activity" OR "physically active" OR "Food" OR "Obesity" OR overweight OR obes*)))	3,621 document results
13	(((TITLE-ABS-KEY ((mhealth OR "mobile health" OR "Telemedicine" OR telehealth OR "Text Messaging" OR "text message" OR "text messages" OR "electronic health"))) AND ((TITLE-ABS-KEY (("Cell Phone" OR "Social Media" OR "mobile phone" OR "mobile phones" OR "cellular phone" OR "cell phones" OR "cellular phones"))) OR ((TITLE-ABS-KEY ((sms OR "Short messaging system" OR "mobile-based interventions" OR computers OR "Hand-held devices" OR pda OR "Personal Digital Assistant" OR tablets OR apps OR "Mobile Applications" OR computer* OR "Pocket PC" OR "Smartphone" OR "Smart phone")) OR TITLE-ABS-KEY (("Smart book" OR iphone OR i-phone OR blackberry OR mms OR "Multimedia messages" OR email OR e-mail OR "Electronic mail" OR web-based OR "Interactive software" OR "Ultra mobile" OR "MP3 player" OR "MP4 player" OR ipod)))))) AND (TITLE-ABS-KEY ((("Diabetes Mellitus, Type 2" OR "diabetes mellitus type 2" OR "type 2 diabetes mellitus" OR ("diabetes" AND "mellitus" AND "type 2")))))) OR (((TITLE-ABS-KEY ((mhealth OR "mobile health" OR "Telemedicine" OR telehealth OR "Text Messaging" OR "text	4,009 document results

History Count	Search Terms	Results
	message" OR "text messages" OR "electronic health"))) AND ((TITLE-ABS-KEY (("Cell Phone" OR "Social Media" OR "mobile phone" OR "mobile phones" OR "cellular phone" OR "cell phones" OR "cellular phones"))) OR ((TITLE-ABS-KEY ((sms OR "Short messaging system" OR "mobile-based interventions" OR computers OR "Hand-held devices" OR pda OR "Personal Digital Assistant" OR tablets OR apps OR "Mobile Applications" OR computer* OR "Pocket PC" OR "Smartphone" OR "Smart phone")) OR TITLE-ABS-KEY (("Smart book" OR iphone OR i-phone OR blackberry OR mms OR "Multimedia messages" OR email OR e-mail OR "Electronic mail" OR web-based OR "Interactive software" OR "Ultra mobile" OR "MP3 player" OR "MP4 player" OR ipod)))))) AND (TITLE-ABS-KEY (("Diet" OR exercise* OR "Physical Activity" OR "physically active" OR "Food" OR "Obesity" OR overweight OR obes*)))) ...View More	
14	(TITLE-ABS-KEY ("African Americans" OR blacks OR "Hispanic Americans" OR "Mexican Americans" OR latino OR latina OR latinx OR "Ethnic Groups" OR "Minority Groups" OR "Population Groups") OR TITLE-ABS-KEY ("Indians, North American" OR "Native American" OR tribal* OR "Asian Americans" OR "Asian American" OR divers* OR "Medically Underserved Area" OR underserved OR "Vulnerable Populations" OR disadvantaged OR "Social Class" OR "low socioeconomic status")) ...View More	2,260,649 document results

History Count	Search Terms	Results
15	((((TITLE-ABS-KEY ((mhealth OR "mobile health" OR "Telemedicine" OR telehealth OR "Text Messaging" OR "text message" OR "text messages" OR "electronic health"))) AND ((TITLE-ABS-KEY (("Cell Phone" OR "Social Media" OR "mobile phone" OR "mobile phones" OR "cellular phone" OR "cell phones" OR "cellular phones"))) OR ((TITLE-ABS-KEY ((sms OR "Short messaging system" OR "mobile-based interventions" OR computers OR "Hand-held devices" OR pda OR "Personal Digital Assistant" OR tablets OR apps OR "Mobile Applications" OR computer* OR "Pocket PC" OR "Smartphone" OR "Smart phone")) OR TITLE-ABS-KEY (("Smart book" OR iphone OR i-phone OR blackberry OR mms OR "Multimedia messages" OR email OR e-mail OR "Electronic mail" OR web-based OR "Interactive software" OR "Ultra mobile" OR "MP3 player" OR "MP4 player" OR ipod)))))) AND (TITLE-ABS-KEY ((("Diabetes Mellitus, Type 2" OR "diabetes mellitus type 2" OR "type 2 diabetes mellitus" OR ("diabetes" AND "mellitus" AND "type 2")))))) OR (((TITLE-ABS-KEY ((mhealth OR "mobile health" OR "Telemedicine" OR telehealth OR "Text Messaging" OR "text message" OR "text messages" OR "electronic health"))) AND ((TITLE-ABS-KEY (("Cell Phone" OR "Social Media" OR "mobile phone" OR "mobile phones" OR "cellular phone" OR "cell phones" OR "cellular phones"))) OR ((TITLE-ABS-KEY ((sms OR "Short messaging system" OR "mobile-based interventions" OR computers OR "Hand-held devices" OR pda OR "Personal Digital Assistant" OR tablets OR apps OR "Mobile Applications" OR computer* OR "Pocket PC" OR "Smartphone" OR "Smart phone")) OR TITLE-ABS-KEY (("Smart book" OR iphone OR i-phone OR blackberry OR mms OR "Multimedia messages" OR email OR e-mail OR "Electronic mail" OR web-based OR "Interactive software" OR "Ultra mobile" OR "MP3 player" OR "MP4 player" OR ipod)))))) AND (TITLE-ABS-KEY (("Diet" OR exercise* OR "Physical Activity" OR "physically active" OR "Food" OR "Obesity" OR overweight OR obes*))))) AND ((TITLE-ABS-KEY ("African Americans" OR blacks OR "Hispanic Americans" OR "Mexican Americans" OR latino OR latina OR latinx OR "Ethnic Groups" OR "Minority Groups" OR "Population Groups") OR TITLE-ABS-KEY ("Indians, North American" OR "Native American" OR tribal* OR "Asian Americans" OR "Asian American" OR divers* OR "Medically Underserved	365 document results

History Count	Search Terms	Results
	Area" OR underserved OR "Vulnerable Populations" OR disadvantaged OR "Social Class" OR "low socioeconomic status"))) ...View More	
16	TITLE-ABS-KEY (((randomized OR randomised) AND controlled AND trial) OR (controlled AND trial) OR "controlled clinical trial" OR "Randomized Controlled Trial" OR "Single-Blind Method" OR "Double-Blind Method" OR "Random Allocation")	1,332,435 document results
17	(((((TITLE-ABS-KEY ((mhealth OR "mobile health" OR "Telemedicine" OR telehealth OR "Text Messaging" OR "text message" OR "text messages" OR "electronic health"))) AND ((TITLE-ABS-KEY (("Cell Phone" OR "Social Media" OR "mobile phone" OR "mobile phones" OR "cellular phone" OR "cell phones" OR "cellular phones"))) OR ((TITLE-ABS-KEY ((sms OR "Short messaging system" OR "mobile-based interventions" OR computers OR "Hand-held devices" OR pda OR "Personal Digital Assistant" OR tablets OR apps OR "Mobile Applications" OR computer* OR "Pocket PC" OR "Smartphone" OR "Smart phone")) OR TITLE-ABS-KEY (("Smart book" OR iphone OR i-phone OR blackberry OR mms OR "Multimedia messages" OR email OR e-mail OR "Electronic mail" OR web-based OR "Interactive software" OR "Ultra mobile" OR "MP3 player" OR "MP4	126 document results

History Count	Search Terms	Results
	player" OR ipod)))))) AND (TITLE-ABS-KEY ((("Diabetes Mellitus, Type 2" OR "diabetes mellitus type 2" OR "type 2 diabetes mellitus" OR ("diabetes" AND "mellitus" AND "type 2")))))) OR (((TITLE-ABS-KEY ((mhealth OR "mobile health" OR "Telemedicine" OR telehealth OR "Text Messaging" OR "text message" OR "text messages" OR "electronic health"))) AND ((TITLE-ABS-KEY (("Cell Phone" OR "Social Media" OR "mobile phone" OR "mobile phones" OR "cellular phone" OR "cell phones" OR "cellular phones"))) OR ((TITLE-ABS-KEY ((sms OR "Short messaging system" OR "mobile-based interventions" OR computers OR "Hand-held devices" OR pda OR "Personal Digital Assistant" OR tablets OR apps OR "Mobile Applications" OR computer* OR "Pocket PC" OR "Smartphone" OR "Smart phone")) OR TITLE-ABS-KEY (("Smart book" OR iphone OR i-phone OR blackberry OR mms OR "Multimedia messages" OR email OR e-mail OR "Electronic mail" OR web-based OR "Interactive software" OR "Ultra mobile" OR "MP3 player" OR "MP4 player" OR ipod)))))) AND (TITLE-ABS-KEY (("Diet" OR exercise* OR "Physical Activity" OR "physically active" OR "Food" OR "Obesity" OR overweight OR obes*))))) AND ((TITLE-ABS-KEY ("African Americans" OR blacks OR "Hispanic Americans" OR "Mexican Americans" OR latino OR latina OR latinx OR "Ethnic Groups" OR "Minority Groups" OR "Population Groups") OR TITLE-ABS-KEY ("Indians, North American" OR "Native American" OR tribal* OR "Asian Americans" OR "Asian American" OR divers* OR "Medically Underserved Area" OR underserved OR "Vulnerable Populations" OR disadvantaged OR "Social Class" OR "low socioeconomic status")))) AND (TITLE-ABS-KEY (((randomized OR randomised) AND controlled AND trial) OR (controlled AND trial) OR "controlled clinical trial" OR "Randomized Controlled Trial" OR "Single-Blind Method" OR "Double-Blind Method" OR "Random Allocation")) ...View More	
18	#17 Limited to studies done in the United States/English language	**96**

Library and Information Source (EBSCOhost)

#	Query	Last Run Via	Results
S1	(mhealth OR "mobile health" OR "Telemedicine" OR telehealth OR "Text Messaging" OR "text message" OR "text messages" OR "electronic health")	Interface - EBSCOhost Research Databases<br>Search Screen - Advanced Search<br>Database - Library & Information Science Source	5,192
S2	("Cell Phone" OR "Social Media" OR "mobile phone" OR "mobile phones" OR "cellular phone" OR "cell phones" OR "cellular phones")	Interface - EBSCOhost Research Databases<br>Search Screen - Advanced Search<br>Database - Library & Information Science Source	17,602
S3	(SMS OR “Short messaging system” OR “mobile-based interventions” OR Computers OR “Hand-held devices” OR PDA OR “Personal Digital Assistant” OR Tablets OR Apps OR "Mobile Applications" OR Computer* OR “Pocket PC” OR "Smartphone" OR “Smart phone” OR “Smart book” OR Iphone OR I-Phone OR Blackberry OR MMS OR “Multimedia messages” OR Email OR E-Mail OR “Electronic mail” OR Web-based OR “Interactive software” OR	Interface - EBSCOhost Research Databases<br>Search Screen - Advanced Search<br>Database - Library & Information Science Source	201,989

	"Ultra mobile" OR "MP3 player" OR "MP4 player" OR Ipod)		
S4	S1 AND (S2 OR S3)	Interface - EBSCOhost Research Databases<br>Search Screen - Advanced Search<br>Database - Library & Information Science Source	3,425
S5	(("Diabetes Mellitus, Type 2" OR "diabetes mellitus type 2" OR "type 2 diabetes mellitus" OR ("diabetes" AND "mellitus" AND "type 2")))	Interface - EBSCOhost Research Databases<br>Search Screen - Advanced Search<br>Database - Library & Information Science Source	198
S6	S4 AND S5	Interface - EBSCOhost Research Databases<br>Search Screen - Advanced Search<br>Database - Library & Information Science Source	58
S7	("Diet" OR Exercise* OR "Physical Activity" OR "physically active" OR "Food" OR "Obesity" OR Overweight OR obes*)	Interface - EBSCOhost Research Databases<br>Search Screen - Advanced Search<br>Database - Library & Information Science Source	30,569

S8	S4 AND S7	Interface - EBSCOhost Research Databases<br>Search Screen - Advanced Search<br>Database - Library & Information Science Source	438
S9	S6 OR S8	Interface - EBSCOhost Research Databases<br>Search Screen - Advanced Search<br>Database - Library & Information Science Source	474
S10	"African Continental Ancestry Group" OR “African Americans” OR Blacks OR “Hispanic Americans” OR “Mexican Americans” OR Latino OR Latina OR Latinx OR “Racial Stocks” OR “Ethnic Groups” OR “Minority Groups” OR “Population Groups” OR “Indians, North American” OR “Native American” OR Tribal* OR “Asian Americans” OR “Asian American” OR divers* OR "Medically Underserved Area" OR underserved OR "Vulnerable Populations" OR disadvantaged OR "Social Class" OR “low socioeconomic status”	Interface - EBSCOhost Research Databases<br>Search Screen - Advanced Search<br>Database - Library & Information Science Source	53,157
S11	S9 AND S10	Interface - EBSCOhost Research Databases	72

		Search Screen - Advanced Search<br>Database - Library & Information Science Source	
S12	S11	Interface - EBSCOhost Research Databases<br>Search Screen - Advanced Search<br>Database - Library & Information Science Source	0
S13	((randomized OR randomised) AND controlled AND trial) OR (controlled AND trial) OR "controlled clinical trial" OR "Randomized Controlled Trial" OR "Single-Blind Method" OR "Double-Blind Method" OR "Random Allocation"	Interface - EBSCOhost Research Databases<br>Search Screen - Advanced Search<br>Database - Library & Information Science Source	2,357
S14	S11 AND S13	Interface - EBSCOhost Research Databases<br>Search Screen - Advanced Search<br>Database - Library & Information Science Source	**23**
